# Poly[μ-(1,3-dihy­droxy­propan-2-olato)-potassium]

**DOI:** 10.1107/S160053681005316X

**Published:** 2011-01-08

**Authors:** Gabriele Schatte, Jianheng Shen, Martin Reaney, Ramaswami Sammynaiken

**Affiliations:** a Saskatchewan Structural Sciences Centre, University of Saskatchewan, Saskatoon, Saskatchewan, Canada S7N 5C9; b Department of Food and Bioresources, University of Saskatchewan, Saskatoon, Saskatchewan, Canada S7N 5A8

## Abstract

The asymmetric unit of the title compound, [K(C_3_H_7_O_3_)]_*n*_ or K[H_2_gl]_*n*_, common name potassium glycerolate, contains half the K^+^ cation and half of the glycerolate anion. The other half of the anion is generated through a mirror plane passing through the K atom, and a C, an H and an O atom of the glycerolate ligand. The K^+^ ion is coordinated by the O atoms of the OH groups, leading to a six-membered chelate ring that adopts a very distorted boat conformation. The negatively charged O atom of the glycerolate anion, [H_2_gl^−^], is found in the flagpole position and forms an ionic bond with the K^+^ ion. The O atoms of the hydroxo groups are coordinated to two K^+^ ions, whereas the negatively charged O atom is bonded to one K^+^ ion. The K^+^ ion is coordinated by three other symmetry-related monodentate H_2_gl^−^ ligands, so that each H_2_gl^−^ ligand is bonded to two K^+^ ions, and the potassium has a seven-coordinate environment. The H_2_gl^−^ ligands are connected *via* a strong O—H⋯O hydrogen bond and, together with the K⋯O inter­connections, form polymeric sheets which propagate in the directions of the *a* and *b* axes.

## Related literature

For syntheses of mono potassium glyceroxide, see: Forcrand (1887 ▶). For syntheses and characterization of potassium alkoxides and aryl­oxides, see: Weiss *et al.* (1968 ▶); Brooker *et al.* (1991 ▶); Kennedy *et al.* (2001 ▶). For the crystal structure of poly[μ-2,3-dihy­droxy­propan-1-olato-sodium], see: Schatte *et al.* (2010 ▶) and for related crystal structures of transition metal mono glyceroxides, see: Rath *et al.* (1998 ▶). 
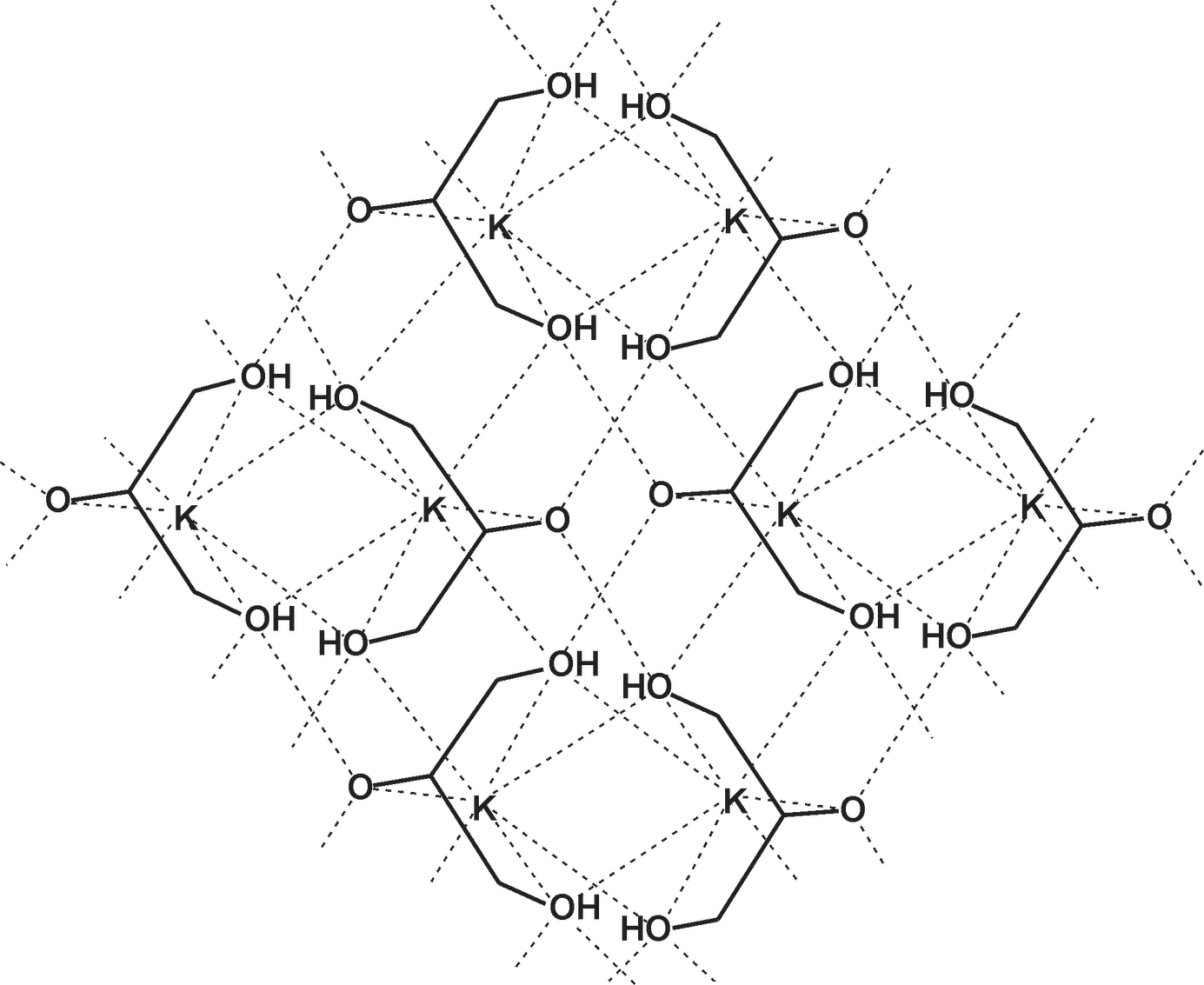


## Experimental

### 

#### Crystal data

[K(C_3_H_7_O_3_)]
*M* *_r_* = 130.19
Monoclinic, 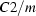

*a* = 7.5514 (4) Å
*b* = 7.2632 (4) Å
*c* = 9.0675 (5) Å
β = 93.422 (2)°
*V* = 496.44 (5) Å^3^
*Z* = 4
Cu *K*α radiation
μ = 8.53 mm^−1^
*T* = 173 K
0.11 × 0.08 × 0.08 mm

#### Data collection

Bruker Proteum R SMART 6000 three-circle diffractometer
Absorption correction: multi-scan (*SADABS*; Bruker, 2008 ▶) *T*_min_ = 0.454, *T*_max_ = 0.549
1526 measured reflections
436 independent reflections
433 reflections with *I* > 2σ(*I*)
*R*_int_ = 0.034

#### Refinement

*R*[*F* ^2^ > 2σ(*F* ^2^)] = 0.043
*wR*(*F* ^2^) = 0.103
*S* = 1.17
436 reflections
41 parameters
H atoms treated by a mixture of independent and constrained refinement
Δρ_max_ = 0.38 e Å^−3^
Δρ_min_ = −0.61 e Å^−3^

### 

Data collection: *APEX2* (Bruker, 2009 ▶); cell refinement: *SAINT* (Bruker, 2008 ▶); data reduction: *SAINT*; program(s) used to solve structure: *SIR2002* (Burla *et al.*, 2003 ▶); program(s) used to refine structure: *SHELXL97* (Sheldrick, 2008 ▶); molecular graphics: *CAMERON* (Watkin *et al.*, 1993 ▶) and *XP* in *SHELXTL-NT* (Sheldrick, 2008 ▶); software used to prepare material for publication: *publCIF* (Westrip, 2010 ▶).

## Supplementary Material

Crystal structure: contains datablocks global, I. DOI: 10.1107/S160053681005316X/om2384sup1.cif

Structure factors: contains datablocks I. DOI: 10.1107/S160053681005316X/om2384Isup2.hkl

Additional supplementary materials:  crystallographic information; 3D view; checkCIF report

## Figures and Tables

**Table:** 

K1—O2	2.690 (2)

**Table:** 

K1⋯O1	2.7726 (16)
K1⋯O1^i^	2.7726 (16)
K1⋯O1^ii^	2.8160 (15)
K1⋯O1^iii^	2.8160 (15)
K1⋯O1^iv^	2.8576 (15)
K1⋯O1^v^	2.8576 (15)

Symmetry codes: (i) 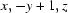; (ii) 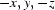; (iii) 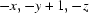; (iv) 
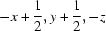
; (v) 
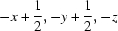
.

**Table 2:** Hydrogen-bond geometry (Å, °)

*D*—H⋯*A*	*D*—H	H⋯*A*	*D*⋯*A*	*D*—H⋯*A*
O1—H1⋯O2^vi^	0.84 (3)	1.68 (3)	2.5229 (19)	177 (3)

Symmetry code: (vi) 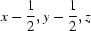.

**Fig. 1.**
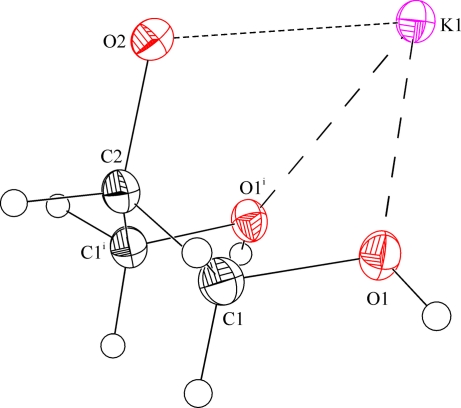
Molecular structure showing the labelling scheme. Non-hydrogen atoms are represented by displacement ellipsoids at the 30% probability level. Symmetry code: (i) x, -y + 1, z.

**Fig. 2.**
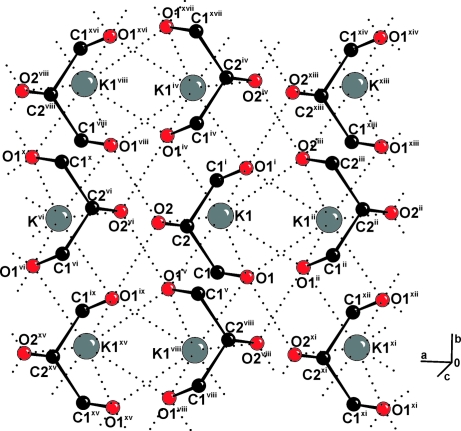
Partial packing diagram of one of the two parallel polymeric sheets observed within the unit cell showing the intra- and inter-molecular K···O and intermolecular O(H)···O contacts (dashed lines). Hydrogen atoms have been omitted for clarity. Symmetry codes: (i) x, -y + 1, z; (ii) -x, y, -z; (iii) -x, -y + 1, -z; (iv) -x + 1/2, y + 1/2, -z; (v) -x + 1/2, -y + 1/2, -z; (vi) -x + 1, y, -z; (vii) -x + 1/2, y - 1/2, -z; (viii) x + 1/2, y + 1/2, z; (ix) x + 1/2, -y + 1/2, z; (x) -x + 1, -y + 1, -z; (xi) x - 1/2, y - 1/2, z; (xii) x - 1/2, -y + 1/2, z; (xiii) x - 1/2, y + 1/2, z; (xiv) x + 3/2, -y + 3/2, z; (xv) x + 1/2, y - 1/2, z; (xvi) x + 1/2, -y + 3/2, z; (xvii) -x + 1/2, -y + 3/2, -z.

## supplementary crystallographic information

### Comment

We have shown that alkali metal glycerolates, although known as alkali metal glyceroxide, can be used as efficient catalysts in *trans*-esterification reactions to produce biodiesel. Earlier syntheses of the mono alkali glycerolate, [*M*(C_3_H_7_O_3_)]*_n_* (referred to as *M*[H_2_gl], *M*= Na, K), involved the reaction of excess alkali metal dissolved in ethanol with glycerol (Forcrand, 1887). We found that adding glycerol to a hot alkali hydroxide solution under agitation is the preferred synthesis for mono alkali glyceroxides (Schatte *et al.*, 2010).

Recently, we have reported the crystal structure of poly[µ-2,3-dihydroxypropan-1-olato-sodium], the until now only known crystal structure of mono alkali glyceroxide. The crystal structure of [K(C_3_H_7_O_3_)]*_n_* (I) was determined as part of our continuing research on catalysts which can be used in the production of biodiesel.

The monodentate H_2_gl^-^ ligand coordinates to the potassium atom *via* the hydroxo groups leading to 6-membered chelating ring with a very distorted boat conformation, Fig. 1. The negatively charged O atom, which is attached to the secondary carbon atom of the H_2_gl^-^ ion, is found in the flagpole position and forms an ionic bond with the potassium cation. This structure is in contrast to the sodium analogue, where the H_2_gl^-^ ligand is coordinated to the sodium atom by one oxo- and one hydroxo group forming a non-planar 5-membered ring and the hydroxo group attached to primary carbon atom of the glycerol is deprotonated. In the related structure of the vanadium-H_2_gl complex, however, the hydroxo group attached to secondary carbon atom is deprotonated as well (Rath *et al.*, 1998).

Each H_2_gl^-^ ligand is bonded to two potassium cations. Each potassium cation is connected *via* K···O bonds ranging from 2.690 (2) to 2.8576 (15) Å to three symmetry related H_2_gl^-^ ligands and is 7-coordinated. In addition, five longer and much weaker K···O interactions ranging from 3.211 (2) to 4.0451 (16) are observed (sum of the van der Waals radii, 4.3 Å; Table 1). The observed intra- and inter-molecular K···O bond distances are elongated in comparison to the related bond distances reported for potassium phenolate complexes (Brooker *et al.*, 1991) and potassium alkoxides (Weiss *et al.*, 1968; Kennedy, *et al.*, 2001).

The H_2_gl^-^ ligands are connected *via* one strong intermolecular O—H···O hydrogen bond interaction (Table 2 and Fig. 2). Both, the K···O and O—H···O interconnections are responsible for the formation of polymeric sheets which extend indefinitely in the directions of the *a* and *b* axes (Fig. 2).

### Experimental

A potassium hydroxide solution (336 g, 50%) was freshly prepared by dissolving potassium hydroxide pellets (168 g, 3 mol) in water (168 g). Glycerol (92 g, 1 mol) was slowly added into the hot potassium hydroxide solution under agitation. The mixture was allowed to stand and to cool down to room temperature. Colourless crystals of Poly[µ-2,3-dihydroxypropan-1-olato-potassium] started to form after six days. The crystals are only stable in a very basic solution at ambient temperatures and are less stable than those of the sodium analogue. A suitable single-crystal was quickly coated with oil, collected onto the aperture of a mounted MiTeGen Micromount^TM^ (diameter of the aperture: 100 microns) and as quickly as possible transferred to the cold stream of the X-ray diffractometer. The crystals tend to dissolve upon eposure to the oil at ambient temperatures for more than one minute.

### Refinement

The C-bound H atoms were geometrically placed (C–H = 0.98–1.00 Å) and refined as riding with *U_iso_*(H) = 1.2*U_eq_*(parent atom). The hydrogen atoms of the hydroxo groups were located in the difference Fourier map and were allowed to refine freely.

### Crystal data

**Table:**

[K(C_3_H_7_O_3_)]	*F*(000) = 272
*M**_r_* = 130.19	*D*_x_ = 1.742 Mg m^−^^3^
Monoclinic, *C*2/*m*	Cu *K*α radiation, λ = 1.54184 Å
Hall symbol: -C 2y	Cell parameters from 1381 reflections
*a* = 7.5514 (4) Å	θ = 4.9–64.3°
*b* = 7.2632 (4) Å	µ = 8.53 mm^−^^1^
*c* = 9.0675 (5) Å	*T* = 173 K
β = 93.422 (2)°	Plate, colourless
*V* = 496.44 (5) Å^3^	0.11 × 0.08 × 0.08 mm
*Z* = 4	

### Data collection

**Table:**

Bruker Proteum R SMART 6000 three-circle diffractometer	436 independent reflections
Radiation source: fine-focus rotating anode	433 reflections with *I* > 2σ(*I*)
Montel200 multilayer graded optics	*R*_int_ = 0.034
Detector resolution: 9 pixels mm^-1^	θ_max_ = 65.0°, θ_min_ = 4.9°
φ and ω scans	*h* = −8→6
Absorption correction: multi-scan (*SADABS*; Bruker, 2008)	*k* = −8→8
*T*_min_ = 0.454, *T*_max_ = 0.549	*l* = −10→10
1526 measured reflections	

### Refinement

**Table:**

Refinement on *F*^2^	Primary atom site location: structure-invariant direct methods
Least-squares matrix: full	Secondary atom site location: difference Fourier map
*R*[*F*^2^ > 2σ(*F*^2^)] = 0.043	Hydrogen site location: inferred from neighbouring sites
*wR*(*F*^2^) = 0.103	H atoms treated by a mixture of independent and constrained refinement
*S* = 1.17	*w* = 1/[σ^2^(*F*_o_^2^) + (0.0682*P*)^2^ + 0.2643*P*] where *P* = (*F*_o_^2^ + 2*F*_c_^2^)/3
436 reflections	(Δ/σ)_max_ < 0.001
41 parameters	Δρ_max_ = 0.38 e Å^−^^3^
0 restraints	Δρ_min_ = −0.61 e Å^−^^3^

### Special details

**Table:**

Geometry. All e.s.d.'s (except the e.s.d. in the dihedral angle between two l.s. planes) are estimated using the full covariance matrix. The cell e.s.d.'s are taken into account individually in the estimation of e.s.d.'s in distances, angles and torsion angles; correlations between e.s.d.'s in cell parameters are only used when they are defined by crystal symmetry. An approximate (isotropic) treatment of cell e.s.d.'s is used for estimating e.s.d.'s involving l.s. planes.
Refinement. Refinement of *F*^2^ against ALL reflections. The weighted *R*-factor *wR* and goodness of fit *S* are based on *F*^2^, conventional *R*-factors *R* are based on *F*, with *F* set to zero for negative *F*^2^. The threshold expression of *F*^2^ > σ(*F*^2^) is used only for calculating *R*-factors(gt) *etc*. and is not relevant to the choice of reflections for refinement. *R*-factors based on *F*^2^ are statistically about twice as large as those based on *F*, and *R*- factors based on ALL data will be even larger.

### Fractional atomic coordinates and isotropic or equivalent isotropic displacement parameters (Å^2^)

**Table:**

	*x*	*y*	*z*	*U*_iso_*/*U*_eq_	
K1	0.20681 (7)	0.5000	−0.05926 (6)	0.0275 (4)	
O1	0.08625 (19)	0.2924 (2)	0.17095 (15)	0.0281 (5)	
H1	0.025 (4)	0.196 (5)	0.182 (3)	0.051 (9)*	
O2	0.4099 (3)	0.5000	0.1948 (2)	0.0256 (6)	
C1	0.1812 (3)	0.3246 (3)	0.3086 (2)	0.0289 (6)	
H1A	0.2604	0.2186	0.3318	0.035*	
H1B	0.0958	0.3329	0.3871	0.035*	
C2	0.2919 (4)	0.5000	0.3097 (3)	0.0265 (7)	
H2	0.3665	0.5000	0.4046	0.032*	

### Atomic displacement parameters (Å^2^)

**Table:**

	*U*^11^	*U*^22^	*U*^33^	*U*^12^	*U*^13^	*U*^23^
K1	0.0261 (5)	0.0302 (5)	0.0258 (5)	0.000	−0.0015 (3)	0.000
O1	0.0288 (8)	0.0286 (9)	0.0264 (9)	−0.0043 (6)	−0.0036 (6)	0.0016 (6)
O2	0.0232 (11)	0.0250 (11)	0.0286 (11)	0.000	0.0012 (9)	0.000
C1	0.0284 (11)	0.0332 (12)	0.0249 (10)	0.0003 (9)	−0.0008 (8)	0.0027 (8)
C2	0.0257 (15)	0.0324 (15)	0.0209 (14)	0.000	−0.0018 (11)	0.000

### Geometric parameters (Å, °)

**Table:**

K1—O2	2.690 (2)	C1—H1A	0.9900
O1—C1	1.421 (3)	C1—H1B	0.9900
O1—H1	0.84 (3)	C2—C1^i^	1.523 (3)
O2—C2	1.411 (4)	C2—H2	1.0000
C1—C2	1.523 (3)		
			
K1···O1	2.7726 (16)	K1···O2^iv^	3.9176 (8)
K1···O1^i^	2.7726 (16)	K1···O2^vii^	3.9176 (8)
K1···O1^ii^	2.8160 (15)	K1···O1^viii^	4.0451 (16)
K1···O1^iii^	2.8160 (15)	K1···O1^ix^	4.0451 (16)
K1···O1^iv^	2.8576 (15)	O1···K1^iii^	2.8160 (15)
K1···O1^v^	2.8576 (15)	O1···K1^v^	2.8576 (15)
K1···O2^vi^	3.211 (2)	O2···K1^x^	3.211 (2)
			
O2—K1—O1^i^	63.34 (5)	O1^xii^—K1—O2^vi^	48.69 (3)
O2—K1—O1	63.34 (5)	O1^xiii^—K1—O2^vi^	48.69 (3)
O1^i^—K1—O1	65.90 (6)	C1—O1—K1	113.60 (12)
O2—K1—O1^xi^	134.71 (5)	C1—O1—K1^iii^	124.89 (12)
O1^i^—K1—O1^xi^	106.01 (4)	K1—O1—K1^iii^	73.99 (4)
O1—K1—O1^xi^	72.13 (5)	C1—O1—K1^xiii^	100.13 (11)
O2—K1—O1^iii^	134.71 (5)	K1—O1—K1^xiii^	85.77 (4)
O1^i^—K1—O1^iii^	72.13 (5)	K1^iii^—O1—K1^xiii^	134.84 (5)
O1—K1—O1^iii^	106.01 (4)	C2—O2—K1	106.22 (15)
O1^xi^—K1—O1^iii^	64.76 (7)	C2—O2—K1^x^	154.98 (16)
O2—K1—O1^xii^	90.44 (4)	K1—O2—K1^x^	98.80 (6)
O1^i^—K1—O1^xii^	94.23 (4)	O1—C1—C2	113.05 (19)
O1—K1—O1^xii^	151.90 (3)	O1—C1—K1^xiii^	55.63 (9)
O1^xi^—K1—O1^xii^	134.84 (5)	C2—C1—K1^xiii^	115.16 (14)
O1^iii^—K1—O1^xii^	84.79 (2)	O1—C1—H1A	109.0
O2—K1—O1^xiii^	90.44 (4)	C2—C1—H1A	109.0
O1^i^—K1—O1^xiii^	151.90 (3)	O1—C1—H1B	109.0
O1—K1—O1^xiii^	94.23 (4)	C2—C1—H1B	109.0
O1^xi^—K1—O1^xiii^	84.79 (2)	H1A—C1—H1B	107.8
O1^iii^—K1—O1^xiii^	134.84 (5)	O2—C2—C1	111.45 (15)
O1^xii^—K1—O1^xiii^	95.99 (6)	O2—C2—C1^i^	111.45 (15)
O2—K1—O2^vi^	81.20 (6)	C1—C2—C1^i^	113.5 (2)
O1^i^—K1—O2^vi^	129.21 (4)	O2—C2—H2	106.7
O1—K1—O2^vi^	129.21 (4)	C1—C2—H2	106.7
O1^xi^—K1—O2^vi^	124.70 (4)	C1^i^—C2—H2	106.7
O1^iii^—K1—O2^vi^	124.70 (4)		
			
O2—K1—O1—C1	10.85 (12)	O1^xii^—K1—O2—C2	132.00 (3)
O1^i^—K1—O1—C1	−60.15 (13)	O1^xiii^—K1—O2—C2	−132.00 (3)
O1^xi^—K1—O1—C1	−177.64 (12)	O2^vi^—K1—O2—C2	180.0
O1^iii^—K1—O1—C1	−121.60 (13)	K1^iii^—K1—O2—C2	0.0
O1^xii^—K1—O1—C1	−11.93 (18)	C1^xiii^—K1—O2—C2	−130.44 (4)
O1^xiii^—K1—O1—C1	99.22 (12)	C1^xii^—K1—O2—C2	130.44 (4)
O2^vi^—K1—O1—C1	61.77 (13)	O1^i^—K1—O2—K1^x^	−142.51 (4)
O2—K1—O1—K1^iii^	132.45 (5)	O1—K1—O2—K1^x^	142.51 (4)
O1^i^—K1—O1—K1^iii^	61.45 (4)	O1^xi^—K1—O2—K1^x^	131.09 (6)
O1^xi^—K1—O1—K1^iii^	−56.04 (5)	O1^iii^—K1—O2—K1^x^	−131.09 (6)
O1^iii^—K1—O1—K1^iii^	0.0	O1^xii^—K1—O2—K1^x^	−48.00 (3)
O1^xii^—K1—O1—K1^iii^	109.68 (7)	O1^xiii^—K1—O2—K1^x^	48.00 (3)
O1^xiii^—K1—O1—K1^iii^	−139.18 (5)	O2^vi^—K1—O2—K1^x^	0.0
O2^vi^—K1—O1—K1^iii^	−176.63 (4)	K1—O1—C1—C2	15.8 (2)
O2—K1—O1—K1^xiii^	−88.36 (4)	K1^iii^—O1—C1—C2	−70.6 (2)
O1^i^—K1—O1—K1^xiii^	−159.37 (2)	K1^xiii^—O1—C1—C2	105.59 (16)
O1^xi^—K1—O1—K1^xiii^	83.14 (3)	K1—O1—C1—K1^xiii^	−89.79 (8)
O1^iii^—K1—O1—K1^xiii^	139.18 (5)	K1^iii^—O1—C1—K1^xiii^	−176.23 (14)
O1^xii^—K1—O1—K1^xiii^	−111.14 (11)	K1—O2—C2—C1	63.95 (18)
O1^xiii^—K1—O1—K1^xiii^	0.0	K1^x^—O2—C2—C1	−116.05 (18)
O2^vi^—K1—O1—K1^xiii^	−37.45 (6)	K1—O2—C2—C1^i^	−63.95 (18)
O1^i^—K1—O2—C2	37.49 (4)	K1^x^—O2—C2—C1^i^	116.05 (18)
O1—K1—O2—C2	−37.49 (4)	K1^x^—O2—C2—K1	180.0
O1^xi^—K1—O2—C2	−48.91 (6)	O1—C1—C2—O2	−55.6 (2)
O1^iii^—K1—O2—C2	48.91 (6)	O1—C1—C2—C1^i^	71.2 (3)

Symmetry codes: (i) *x*, −*y*+1, *z*; (ii) −*x*, *y*, −*z*; (iii) −*x*, −*y*+1, −*z*; (iv) −*x*+1/2, *y*+1/2, −*z*; (v) −*x*+1/2, −*y*+1/2, −*z*; (vi) −*x*+1, *y*, −*z*; (vii) −*x*+1/2, *y*−1/2, −*z*; (viii) *x*+1/2, *y*+1/2, *z*; (ix) *x*+1/2, −*y*+1/2, *z*; (x) −*x*+1, −*y*+1, −*z*; (xi) −*x*, *y*, −*z*; (xii) −*x*+1/2, *y*+1/2, −*z*; (xiii) −*x*+1/2, −*y*+1/2, −*z*.

### Hydrogen-bond geometry (Å, °)

**Table:**

*D*—H···*A*	*D*—H	H···*A*	*D*···*A*	*D*—H···*A*
O1—H1···O2^xiv^	0.84 (3)	1.68 (3)	2.5229 (19)	177 (3)

Symmetry codes: (xiv) *x*−1/2, *y*−1/2, *z*.
